# Fabrication of Micro-Groove on the Surface of CFRP to Enhance the Connection Strength of Composite Part

**DOI:** 10.3390/polym13224039

**Published:** 2021-11-22

**Authors:** Bin Xu, Meng-Yang Wei, Xiao-Yu Wu, Lian-Yu Fu, Feng Luo, Jian-Guo Lei

**Affiliations:** 1 Shenzhen Key Laboratory of High Performance Nontraditional Manufacturing, Shenzhen University, 3688 Nanhai Avenue, Nanshan District, Shenzhen 518060, China; binxu@szu.edu.cn (B.X.); 2070292024@email.zu.cn (M.-Y.W.); wuxy@szu.edu.cn (X.-Y.W.); LLF@szu.edu.cn (F.L.); 2 Shenzhen Jinzhou Precision Technology Corp., Shenzhen 518060, China; mlyfu@chinadrill.com

**Keywords:** carbon fiber composite, polyamide 6, composite part, micro-groove, connection strength

## Abstract

Carbon fiber-reinforced plastic (CFRP) has the advantages of being light weight, high strength, and corrosion resistant. At present, it is widely used in the lightweight design of automobile parts. The manufacturing of lightweight parts inevitably involves the connection between CFRP and the polymer material. The connection strength between CFRP and the polymer material significantly affects the service life of the composite parts. Taking CFRP and polyamide 6 (PA6) injection-molded composite parts as an example, this paper proposed a technological method to enhance the connection strength between CFRP and PA6. The proposed method was to fabricate micro-groove structures on the CFRP surface by compression molding. These micro-groove structures effectively increased the injection-molding area of the composite parts, thus enhancing the connection strength between CFRP and PA6. This paper presented a detailed study on the compression-molding process of micro-grooves on the CFRP surface, and successfully obtained the appropriate parameters. Finally, PA6 was used for injection molding on the CFRP with micro-grooves at an injection pressure of 8 MPa, an injection temperature of 240 °C, a holding pressure of 5 MPa, and a holding time of 2.5 s. The experimental results show that the micro-groove array structures on the CFRP surface could effectively improve the tensile strength of the connection interface in the composite parts. Compared with the composite part without micro-grooves, the tensile strength of the composite part with micro-grooves was increased by 80.93%. The composite parts prepared in this paper are mainly used in automobile interiors and the research results of this paper meet the actual needs of the enterprise.

## 1. Introduction

In order to achieve the goal of energy saving and emission reduction in fuel vehicles, lightweight vehicles have become the trend in automotive development around the world. Carbon fiber-reinforced plastic (CFRP) has the advantages of being light weight, high strength, and corrosion resistant, and can improve the properties and performances of many industrial parts by replacing conventional metal-based materials [1,2]. Based on these advantages, CFRP has been widely used in the lightweight design of automobile parts. The manufacturing of lightweight parts inevitably involves the connection between CFRP and the polymer material. Different material connections can be mechanical [3], adhesive [4], or molten welding connections [5]. CFRP and the polymer material can be used to produce composite parts using the injection-molding process, which is an excellent approach for producing low-cost and highly repeatable composite parts used in various fields, such as IT, healthcare, and the biomedical sector [6,7,8]. Obviously, the connection strength between the above two materials has an important influence on the service life of the composite parts.

Grzegorz et al. [9,10,11] added the composites to the cement matrix and studied the impact on the fracture toughness of the concrete in order to save energy. Li et al. [12] fabricated PA6- and carbon fiber-reinforced thermoplastic composites through vacuum-assisted resin transfer molding. Moreover, they also studied the effect of temperature on the mechanical properties and polymerization kinetics of PA6. By using a compression-molding process, Zolfaghari et al. [13] used polymethylmethacrylate to fabricate a microlens array. By using a micro hot embossing process, Wang et al. [14] adopted polycarbonate to fabricate polymeric micro-structures and they also investigated polymer recovery of polymeric micro-structures. To form fine patterns arbitrarily, Yun et al. [15] proposed a new hot embossing technology based on the concept of a dot or line printer. In order to fabricate micro-lens array, Li et al. [16] adopted polymethyl methacrylate in the micro-embossing process by using a micro-electrical discharge machining mold.

Focus on the rapid and flexible fabrication of micro-structure patterns, Chang et al. [17] proposed a multi-layered hot embossing method that involved a multi-layered hot embossing process with three polymer substrates. To enhance the mechanical properties of wood–plastic composites (WPC), Guo et al. [18] applied direct injection molding to produce the hybrid WPC with glass fiber (GF) and carbon fiber (CF). By using a micro-milled aluminum mold, Çoğun et al. [19] fabricated micro-fluidic channels through hot embossing and they also investigated the effect of the main process parameters on the processing quality. Superhydrophobic surfaces are widely used as an oil–water separator. Focusing on this, Moon et al. [20] used a hot imprinting process to fabricate a superhydrophobic surface with a large area. By using the titanium master as a mold insert, Ristok et al. [21] achieved the mass production compatible fabrication of polymer-based micro-lenses using injection compression molding.

The above research enabled progress in the molding process of polymer materials and CFRP. However, there are few reports on enhancing the connection of the abovementioned two materials using the injection-molding process. The connection strength between CFRP and the polymer material significantly affects the service life of composite parts. In this paper, in order to enhance the connection strength between them, micro-groove structures were fabricated on the CFRP surface by compression molding. These micro-groove structures were able to effectively increase the injection-molding area of composite parts, thus enhancing the connection strength between CFRP and the polymer material.

## 2. Technological Process

**Figure 1:**
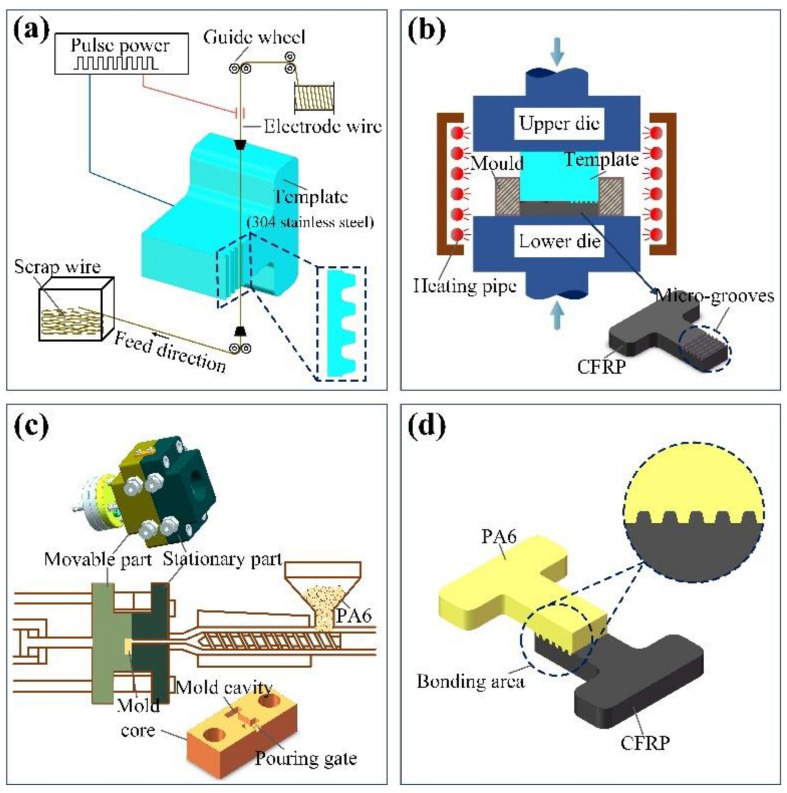
The process flow of composite part preparation: (**a**) fabrication of micro-groove using LS-WEDM; (**b**) compression molding; (**c**) injection molding; (**d**) composite part.

The process flow of injection molding composite parts of CFRP and the polymer material is described as follows:

(1) Micro-groove structures were processed on 304 stainless steel surfaces by low-speed wire electrical discharge machining (LS-WEDM) (Figure 1a).
(2) By using 304 stainless steel surfaces with micro-grooves as a template, micro-grooves were fabricated on the CFRP surface using the compression-molding process (Figure 1b).
(3) This paper installed the CFRP with micro-groove structures in an injection mold and used a polymer material for injection molding (Figure 1c), so as to obtain the composite part (Figure 1d).

The composite parts obtained using the above process possess the following advantages. (1) The micro-groove structures on the CFRP surface can significantly increase the contact area between materials, thus enhancing the connection strength between CFRP and the polymer material. (2) The micro-grooves on the CFRP surface are embedded into the polymer material like nails, thus further improving the connection strength of the connection interface in the composite part.

## 3. Experimental Materials and Equipment

The template in the compression-molding process was made of 304 stainless steel surfaces with a thickness of 5 mm. The polymer material used in the injection molding of composite parts was polyamide 6 (PA6). The physical properties of PA6 are shown in Table 1.

CFRP was provided by Shenzhen Silver Basis Technology Co., Ltd. The tensile strength, tensile modulus, density of the carbon fiber, and content of PA6 were 4.3 GPa, 230 GPa, 1.78 g/cm^3^, and 1%~5%, respectively. The content of the fiber in the CFRP was tested using a thermal gravimetric analyzer (TGA55, TA Instruments, New Castle, DE, USA) and the content of the fiber was 67.14%. An LS-WEDM machine tool (AP250L, Sodick, Yokohama, Japan) was used to fabricate a micro-groove array with a depth of 180 μm on 304 stainless steel plates. The micro-groove had a bottom dimension of 53 μm, and an inclination angle of 12°. The 304 stainless steel plates with micro-groove array structures were installed in a molding press (SZU1.0, Shenzhen University, Shenzhen, China), and micro-groove array structures were fabricated on the CFRP surface by compression molding. The CFRP with micro-groove structures was installed in a micro-injection molding machine (Babyplast 6/10P, Cronoplast Sl, Barcelona, Spain), and the PA6 was used for injection molding, so as to obtain composite parts. The micro-groove array structures on the CFRP surface were examined with a laser confocal microscope (VK250, KEYENCE, Osaka, Japan) to obtain a 3D diagram and cross-sectional profile. The tensile strength of the composite parts was examined using a universal tensile machine (Z050TEW, ZWICK, Ulm, Germany).

## 4. Compression Molding of Micro-Groove on the CFRP Surface

CFRP has the advantages of light weight, high strength and corrosion resistant. The CFRP processing technologies include the injection-molding process, the lamination-molding process, the filament winding process, the pultrusion-molding process, and the compression-molding process [22]. The compression-molding process is a common technology for processing CFRP and it has been widely used in industrial production [23]. In the compression-molding process, CFRP was placed on the template and pressed at a high temperature and high pressure for a certain period to obtain CFRP products with specific shape characteristics. The compression-molding process has advantages such as high machining accuracy, low environmental impact, and so on. In this paper, the effects of temperature, pressure, and holding time on the molding quality of micro-grooves were studied.

### 4.1. Effect of Temperature on the Molding Quality of Micro-Grooves

The molding temperature can soften the CFRP in the template cavity, so as to obtain sufficient fluidity. Therefore, temperature is an extremely important process parameter in the compression-molding process. In order to obtain an appropriate temperature, different molding temperatures were applied to form micro-grooves on the CFRP surface in this paper. The pressure was 300 MPa and the holding time was 10 min. To avoid material degradation due to excessive temperatures, the molding temperatures were set to 120 °C, 140 °C, 160 °C, and 180 °C respectively.

**Figure 2:**
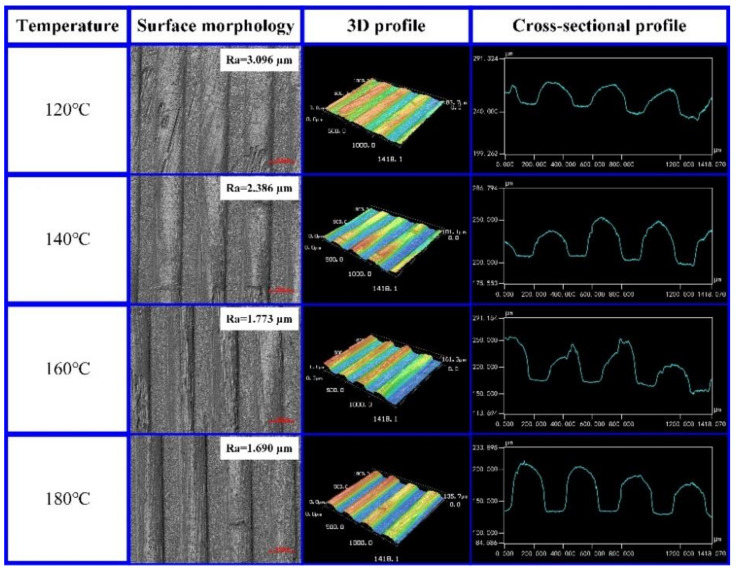
Effect of temperature on the molding quality of the micro-grooves on the CFRP surface.

The experimental results were tested by laser confocal microscopy. The surface morphology, 3D profile, and cross-sectional profile of the micro-groove on the CFRP surface were obtained. The experimental results are shown in Figure 2. According to the experimental results, with the increase in the molding temperature, the softening degree of the CFRP increased, and the molding quality and 3D profile of the micro-grooves improved. When the molding temperature increased gradually from 120 °C to 180 °C, the cross-sectional profile of the micro-grooves was increasingly closer to the micro-structures on the template, and the surface roughness of the micro-grooves gradually decreased from 3.096 μm to 1.690 μm. The replication rate of the micro-grooves on the CFRP surface was tested with a laser confocal microscope. The test results showed that the replication rate of the micro-grooves on the CFRP surface increased from 9.22% to 40.26% as the molding temperature increased from 120 °C to 180 °C.

CFRP exhibits three mechanical states at different temperatures, which are glass state, a high-elastic state, and a viscous flow state. The glass transition temperature *Tg* refers to the temperature corresponding to the transition of materials from the glass state to the high-elastic state, which directly affects the service performance and process performance of materials. Below the glass transition temperature *Tg*, CFRP is in a passivated state. The CFRP in the passivated state has a high elastic modulus and stiffness, but poor molding ability. Above the glass transition temperature *Tg*, CFRP is in a soft state. The CFRP in the soft state has a good stability, elasticity, and a good molding ability. When the temperature is above a certain threshold, CFRP is in the viscous flow state. CFRP in this state will undergo irreversible deformation, which will adversely affect the molding quality.

**Figure 3:**
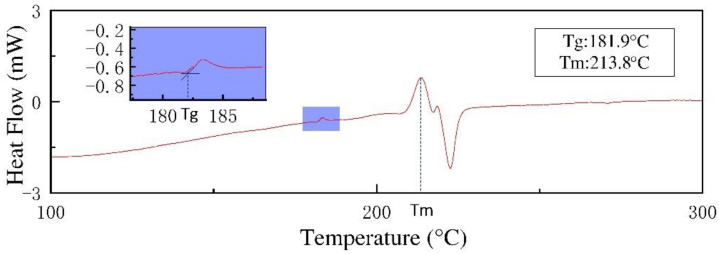
DSC testing of the CFRP.

DSC tests were performed on the CFRP to obtain the glass transition temperature *Tg*, and the test results are shown in Figure 3. According to the experimental results, the glass transition temperature *Tg* of CFRP was 181.9 °C. When the molding temperature was 120 °C, 140 °C, and 160 °C, CFRP exhibited poor plasticity, so the surface micro-grooves had a poor molding quality and a low replication rate (Figure 2). When the molding temperature was 180 °C, the molding temperature was close to the glass transition temperature of CFRP and CFRP had better plasticity and stability. Under the action of molding pressure, the micro-groove structures were gradually replicated on the CFRP surface. Under this working condition, the surface micro-groove structures had a better molding quality and replication rate (Figure 2).

According to the above analysis, in order to ensure the molding quality of the micro-grooves on the CFRP surface, the molding temperature should be close to the glass transition temperature of CFRP. If the molding temperature is too low, CFRP will have poor plasticity, resulting in poor molding quality and a low micro-groove replication rate. If the molding temperature is too high, CFRP will be seriously deformed, resulting in poor molding quality and a low micro-groove replication rate. Therefore, by comparing the experimental results, the molding temperature of CFRP was set to 180 °C in this paper.

### 4.2. Effect of Pressure on the Molding Quality of Micro-Grooves

Under the action of molding pressure, the softened CFRP was gradually filled into the micro-groove structure, thus completing the replication of the surface micro-groove structures. The molding pressure can promote the movement of molecules within the CFRP, which facilitates the replication of micro-groove structures. Therefore, the molding pressure has an important influence on the molding quality of micro-groove structures. In order to obtain an appropriate molding pressure, different molding pressures were applied to form micro-grooves on the CFRP surface. The temperature was 180 °C and the holding time was 10 min. The pressures were set to 200 MPa, 250 MPa, 300 MPa, 350 MPa, 400 MPa, and 450 MPa, respectively.

**Figure 4:**
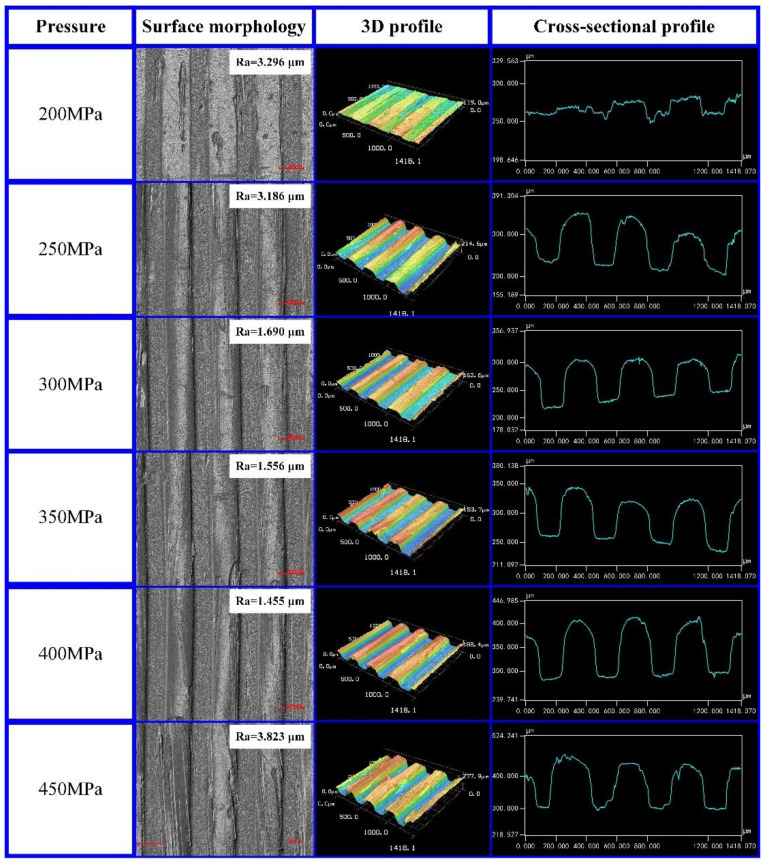
Effect of pressure on the molding quality of the micro-grooves on the CFRP surface.

The micro-grooves on the CFRP surface were tested with a laser confocal microscope to obtain the surface morphology, 3D profile, and cross-sectional profile. The experimental results are shown in Figure 4. According to the experimental results, with the increase in molding pressure, the rate of softening of the carbon fiber composite filling the micro-groove structure increased gradually, and the molding quality and 3D profile of micro-grooves on the CFRP surface were continuously improving. When the molding pressure increased gradually from 200 MPa to 400 MPa, the cross-sectional profile of the micro-grooves became closer to the micro-structure on the template. At this time, the surface roughness of the micro-grooves gradually decreased from 3.296 μm to 1.455 μm. When the pressure was 450 MPa, the surface roughness of the micro-grooves was the greatest (*Ra* = 3.823 μm). The replication rate of micro-grooves on the CFRP surface was examined with a laser confocal microscope. As the pressure gradually increased from 200 MPa to 400 MPa, the replication rate of micro-grooves gradually increased from 9.05% to 62.61%. When the pressure was 450 MPa, the replication rate of micro-structures on the CFRP surface was 54.87%.

Molding pressure can cause the template and the surface of the CFRP to fit closely together, thus facilitating the heat conduction and molding of CFRP. Under the action of molding pressure, the shear deformation and shear rate of CFRP increased, and some molecular chains were broken, which led to the decrease in molecular weight of the CFRP. The relationship between molecular weight and viscosity of CFRP can be expressed by Equation (1):

(1) $$\eta_{0}=AM_{w}^{3.4}$$

where $\eta_{0}$ denotes the apparent viscosity at a lower shear rate; *A* is the empirical constant; and $M_{w}$ denotes the average molecular weight.

According to Equation (1), the viscosity of CFRP is directly proportional to its molecular weight. Under the effect of molding pressure, the molecular weight of CFRP decreased, which led to the decrease in its viscosity. The lower the viscosity of CFRP, the better its fluidity. Therefore, as the molding pressure gradually increased from 200 MPa to 400 MPa, the replication rate of the micro-structures on the CFRP surface became higher and higher. With the further increase in molding pressure, the shear deformation of the CFRP also increased. The shearing action increased the chance of collision of molecules in the CFRP, thus reducing the activation energy of the intermolecular reaction and increasing the rate of the intermolecular cross-linking reaction. Under the action of the cross-linking reaction, the internal molecules of CFRP were linked with each other, which led to the increase in their molecular weight. Under the influence of the above factors, the viscosity of CFRP increased and the fluidity decreased, which eventually led to the decline in the molding ability of the CFRP. Therefore, when the pressure was 450 MPa, the replication rate and molding quality of the micro-structures on the CFRP surface decreased.

According to the above analysis, in order to ensure the molding quality of the micro-grooves on the CFRP surface, the molding pressure should be in an appropriate range. If the molding pressure is too low, CFRP will have poor fluidity, resulting in poor molding quality and a low micro-groove replication rate. If the molding pressure is too high, the viscosity of CFRP will increase, resulting in poor molding quality and a low micro-groove replication rate. Therefore, by comparing the experimental results, the molding pressure of CFRP was set to 400 MPa in this paper.

### 4.3. Effect of Holding Time on the Molding Quality of Micro-Grooves

The holding time refers to the period in which the CFRP material and the template are kept in close contact under the condition that the molding temperature and molding pressure remain unchanged. The holding time has an important influence on the molding quality of micro-grooves. If the holding time is too short, the molding quality will be poor. If the holding time is too long, the processing efficiency will be reduced. In order to obtain an appropriate holding time, different holding times were applied to form micro-grooves on the CFRP surface in this paper. The temperature was 180 °C and the holding pressure was 400 MPa. The holding time periods were 4 min, 6 min, 8 min, 10 min, 12 min, and 14 min, respectively.

**Figure 5:**
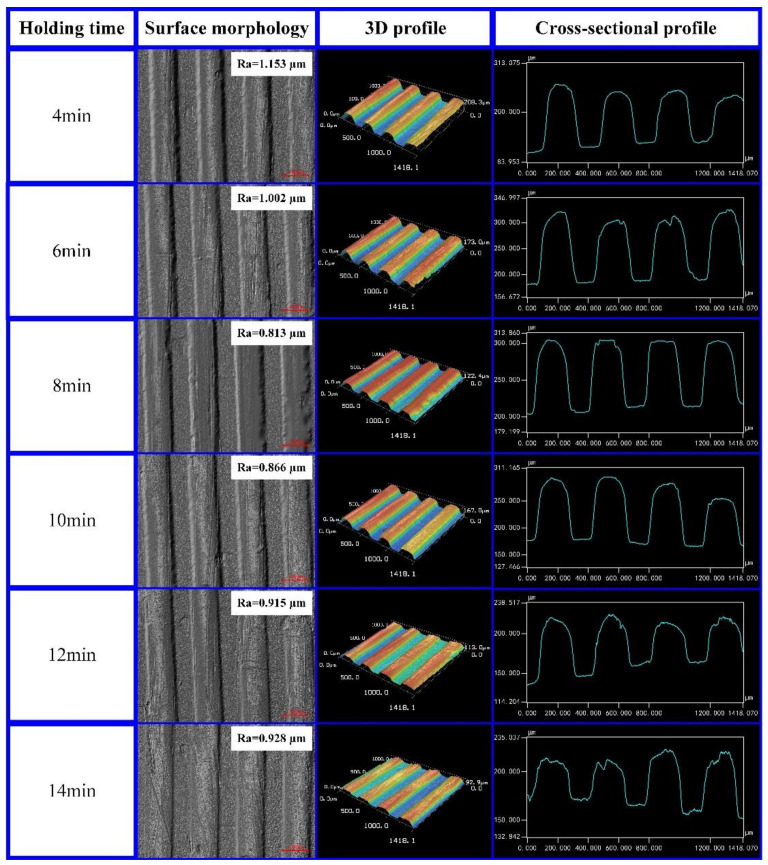
Effect of holding time on the molding quality of the micro-grooves on the CFRP surface.

The experimental results were examined with a laser confocal microscope to obtain the surface morphology, 3D profile, and cross-sectional profile of the micro-grooves on the CFRP surface. The experimental results are shown in Figure 5. It can be seen from the experimental results that as the holding time gradually increased from 4 min to 8 min, the cross-sectional profile of the micro-grooves was increasingly closer to the micro-structures on the template. At this time, the surface roughness of the micro-grooves gradually decreased from 1.153 μm to 0.813 μm. As the holding time gradually increased from 10 min to 14 min, the cross-sectional profile of the micro-grooves became worse. At this time, the surface roughness of the micro-grooves gradually increased from 0.866 μm to 0.928 μm. When the holding time was 8 min, the cross-sectional profile and molding quality of the micro-grooves were better, and the surface roughness was the smallest (*Ra* = 0.813 μm). The replication rate of the micro-grooves on the CFRP surface was examined with a laser confocal microscope. As the holding time increased from 4 min to 8 min, the replication rate of the micro-grooves gradually increased from 62.91% to 65.64%. As the holding time increased from 10 min to 14 min, the replication rate of the micro-grooves decreased from 62.61% to 31.11%.

At the initial stage of the holding time period, some molecular chains of CFRP broke under the action of molding pressure, which led to the decrease in the molecular weight of CFRP. The lower the molecular weight of CFRP, the lower its viscosity. Therefore, at the initial stage of the holding time period, the fluidity and formability of CFRP were better. Therefore, when the holding time was less than 8 min, the molding quality and replication rate of surface micro-grooves gradually increased with the increase in the holding time. As the holding time increased from 8 min to 14 min, the cross-linking reaction of molecules in the CFRP gradually dominated. Under the action of the cross-linking reaction, the internal molecules of CFRP were linked with each other, which led to the increase in their molecular weight. Under the influence of the above factors, the viscosity of the CFRP increased and the fluidity decreased, which eventually led to the decline in the molding ability of the CFRP. Therefore, too long a holding time will adversely affect the molding quality of surface micro-grooves.

According to the above analysis, in order to ensure the molding quality of micro-grooves on the CFRP surface, the holding time should be maintained within an appropriate range. If the holding time is too long, the viscosity of the CFRP will increase, resulting in poor molding quality and a low micro-groove replication rate. Therefore, by comparing the experimental results, the holding time of the CFRP was set to 8 min in this paper.

## 5. Injection Molding of Composite Parts

In this paper, PA6 was used for injection molding micro-grooves onto the CFRP. The $\bar{M}$w of PA6 is 2.45~3.29 × 10^4^ and the $\bar{M}$n of PA6 is 1.46~1.98 × 10^4^. The winding degree of the molecular chain in PA6 is good, which mean that PA6 has good mechanical properties and is heat resistant. In addition, the α value of the molecular weight distribution of PA6 is 1.45~1.76. Under the influence of this factor, PA6 has better processing characteristics and thus its injection-molded parts are more uniform.

### 5.1. Effect of the PA6 Crystal Properties on the Tensile Strength of the Composite Parts

**Figure 6:**
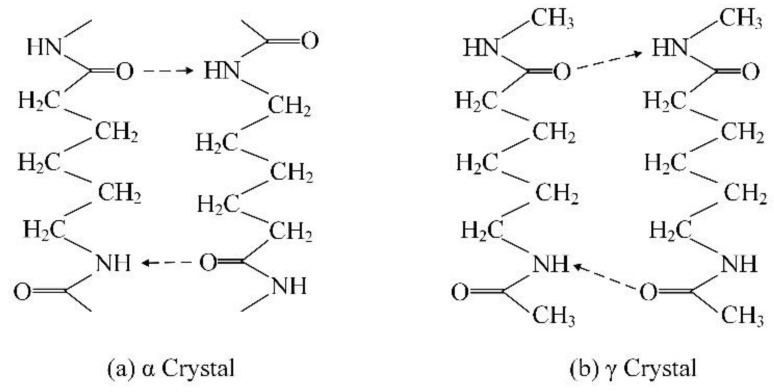
Crystal structures of the α crystal type and the γ crystal type.

PA6 is a typical crystalline polymer. During the injection-molding process, different injection temperatures can result in different crystal structures in PA6. The most common crystal structures in PA6 are the α crystal type and γ crystal type [24,25]. The α crystal type and γ crystal type have different arrangements of molecular chains in PA6. The structural diagrams of the α crystal type and γ crystal type are shown in Figure 6. The molecular chain is fully extended in the α crystal and the polymer chain is in a reverse parallel orientation. The α crystal has a high proportion of hydrogen bonds and the molecular chains in the crystal region are closely arranged. Under the influence of the above factors, the α crystal has high thermodynamic stability and high mechanical strength. The γ crystal chains are arranged in parallel and it has a low proportion of hydrogen bonds. Consequently, the γ crystal has low thermodynamic stability and mechanical strength. In the process of CFRP- and PA6 injection-molded composite parts, different injection temperatures can lead to different crystal forms in PA6. Based on the above analysis, the α crystal can cause the composite parts to have better mechanical properties.

**Figure 7:**
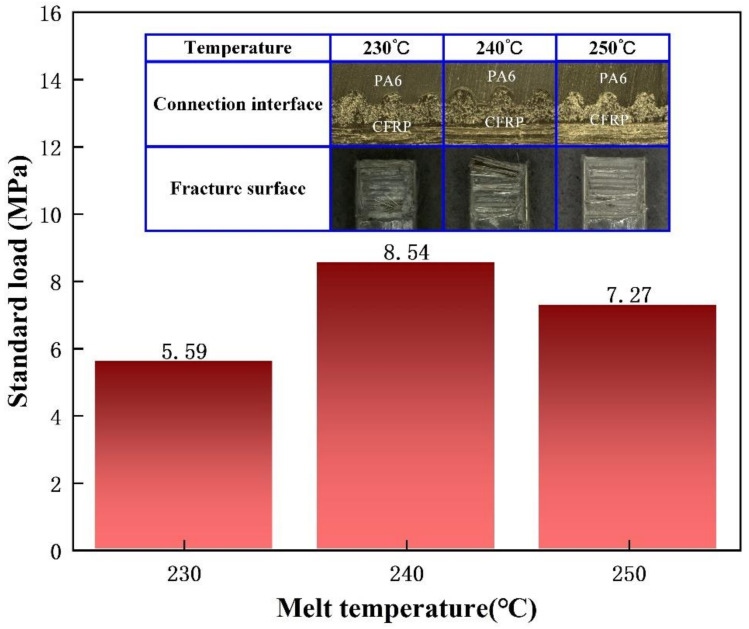
Effect of melt temperatures on the tensile strength of the composite parts.

In order to obtain suitable temperature parameters, different injection temperatures were applied in the injection-molding process of the composite parts. The injection pressure was 8 MPa, the holding pressure was 5 MPa, and the holding time was 2.5 s. The injection temperatures were 230 °C, 240 °C, and 250 °C, respectively. The tensile strength of the composite parts was tested using a universal tensile machine. The experimental results are shown in Figure 7. When the injection temperature increased from 230 °C to 240 °C, the tensile strength increased from 5.59 MPa to 8.54 MPa. When the injection temperature increased from 240 °C to 250 °C, the tensile strength decreased from 8.54 MPa to 7.27 MPa. Therefore, the composite parts demonstrated a good injection molding quality and high tensile strength when the injection temperature was 240 °C.

In the injection-molding process of CFRP and PA6 composite parts, the injection temperature can affect the crystal type in PA6. When the injection temperature was low, it was not conducive to the formation of α crystals. At this point, the crystal structure of injection-molded PA6 was mainly composed of γ crystals, which resulted in the low tensile strength of the composite part. However, when the injection temperature was too high, the hydrogen bonds in PA6 broke, which led to the degradation of the polymer material. In this case, the composite part had a poor injection molding quality and low tensile strength. Based on the experimental results shown in Figure 7, it is suitable to set the injection temperature at 240 °C. Under the injection temperature of 240 °C, the crystal structure of injection-molded PA6 was mainly composed of α crystals, which can effectively guarantee the mechanical properties of the composite part. In addition, the fibers in the CFRP acted as nucleating agents, which can promote the crystallization process of PA6 molecules and accelerate the crystallization rate. Under the influence of the above factors, the injection-molded PA6 had better mechanical properties and it was ultimately improved the molding quality and tensile strength of the composite parts [26].

### 5.2. Comparative Experiments with Composite Parts without Micro-Grooves

**Figure 8:**
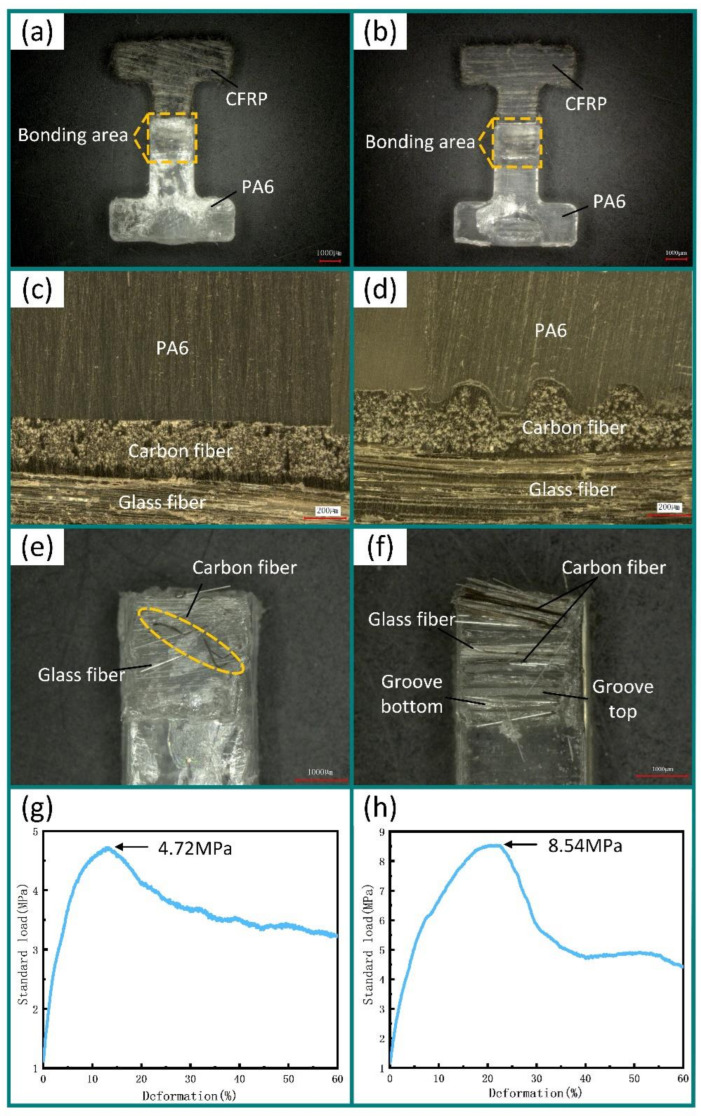
(**a**) Composite part without micro-grooves; (**b**) composite part with micro-grooves; (**c**) connection interface of the composite part without micro-grooves; (**d**) connection interface of the composite part with micro-grooves; (**e**) tensile fracture surface of the composite part without micro-grooves; (**f**) tensile fracture surface of the composite part with micro-grooves; (**g**) tensile strength of the composite part without micro-grooves; (**h**) tensile strength of the composite part with micro-grooves.

In this paper, two experiments were used for comparison. In Experiment 1, CFRP without micro-groove structures was used for the injection molding of the composite parts. In Experiment 2, the micro-groove array structures on the CFRP surface were fabricated by compression molding at a molding pressure of 400 MPa, a molding temperature of 180 °C, and a holding time of 8 min. The cross-sectional profile of micro-grooves was a beveled surface with an inclination angle of 12°. The micro-groove had a bottom dimension of 53 μm and a depth of 180 μm. PA6 was used for injection molding on the CFRP in Experiment 1 and Experiment 2. The injection pressure was 8 MPa, the injection temperature was 240 °C, the holding pressure was 5 MPa, and the holding time was 2.5 s. The experimental results are shown in Figure 8a,b.

Composite parts obtained from Experiment 1 and Experiment 2 were polished (Figure 8c,d). The experimental results showed that CFRP and PA6 formed a good connection in the composite part. Tensile tests were carried out on the composite parts obtained from Experiment 1 and Experiment 2. Then, their fracture surfaces were observed (Figure 8e,f). Compared with the composite part obtained in Experiment 1, there was a lot of CFRP on the tensile fracture surface of the composite part obtained in Experiment 2 (Figure 8f). The maximum tensile strength of the composite part obtained in Experiment 1 was 4.72 MPa, and that of the composite part obtained by Experiment 2 was 8.54 MPa. The experimental results indicate that the micro-groove array structures on the CFRP surface effectively improve the tensile strength of the connection interface in composite parts. Compared with Experiment 1, the tensile strength of composite part obtained in Experiment 2 was improved by 80.93%.

## 6. Conclusions

The connection strength between CFRP and PA6 significantly affects the service life of composite parts. In order to enhance the connection strength between CFRP and PA6, micro-groove array structures were fabricated on the CFRP surface using the compression-molding process, and the CFRP obtained in this process was used for the injection molding of composite parts. The main conclusions of this paper are as follows:

(1) The micro-groove array structures were fabricated on the CFRP surface by compression molding at a molding pressure of 400 MPa, a molding temperature of 180 °C, and a holding time of 8 min. The micro-groove array structures demonstrated a good surface morphology, and the replication rate reached 65.64%.
(2) PA6 was used for the injection molding of micro-grooves on the CFRP at an injection pressure of 8 MPa, an injection temperature of 240 °C, a holding pressure of 5 MPa, and a holding time of 2.5 s. The micro-groove array structures on the CFRP surface effectively improved the tensile strength of the connection interface in the composite parts. Compared with the composite part without micro-grooves, the tensile strength of the composite part with micro-grooves was increased by 80.93%.
(3) Compared with molding pressure and molding temperature, the effects of holding time on the molding quality of the micro-grooves on the CFRP surface were more significant. When the holding time was set to 8 min, the minimum surface roughness *Ra* at the groove bottom of the micro-structured CFPR was 0.813 µm.

## Figures and Tables

**Table 1:** The physical properties of the PA6.

Density (g/cm^3^)	Melting Point (°C)	Bending Strength (MPa)	Water Absorption (%)
1.13	215	90.0	3.5

## Data Availability

The data presented in this study are available on request from the corresponding author.

## References

[1] Hwang D., Lee S.G., Cho D. (2021). Dual-Sizing Effects of Carbon Fiber on the Thermal, Mechanical, and Impact Properties of Carbon Fiber/ABS Composites. Polymers.

[2] Go S.-H., Tugirumubano A., Kim H.-G. (2021). Analysis of Impact Characteristics and Detection of Internal Defects for Unidirectional Carbon Composites with Respect to Fiber Orientation. Polymers.

[3] Lambiase F. (2015). Joinability of different thermoplastic polymers with aluminium AA6082 sheets by mechanical clinching. Int. J. Adv. Manuf. Technol..

[4] Ni J., Min J., Wan H., Lin J., Wang S., Wan Q. (2019). Effect of adhesive type on mechanical properties of galvanized steel/SMC adhesive-bonded joints. Int. J. Adhes. Adhes..

[5] Balle F., Wagner G., Eifler D. (2010). Ultrasonic spot welding of aluminum sheet/carbon fiber reinforced polymer–joints. Mater. Und Werkst..

[6] Lu Y.J., Luo W., Wu X.Y., Xu B., Wang C.J., Li J.J., Li L.J. (2020). Fabrication of Micro-Structured LED Diffusion Plate Using Efficient Micro Injection Molding and Micro-Ground Mold Core. Polymers.

[7] Surace R., Basile V., Bellantone V., Modica F., Fassi I. (2021). Micro Injection Molding of Thin Cavities Using Stereolithography for Mold Fabrication. Polymers.

[8] Ahmad M., Waseem M. (2020). Effects of injection molding parameters on cellular structure of roofing tiles composite. Mater. Today Proc..

[9] Szostak B., Golewski G.L. (2020). Improvement of Strength Parameters of Cement Matrix with the Addition of Siliceous Fly Ash by Using Nanometric C-S-H Seeds. Energies.

[10] Golewski G.L., Gil D.M. (2021). Studies of Fracture Toughness in Concretes Containing Fly Ash and Silica Fume in the First 28 Days of Curing. Materials.

[11] Golewski G.L. (2020). Energy Savings Associated with the Use of Fly Ash and Nanoadditives in the Cement Composition. Energies.

[12] Li M.-X., Lee D., Lee G.H., Kim S.M., Ben G., Lee W.I., Choi S.W. (2020). Effect of Temperature on the Mechanical Properties and Polymerization Kinetics of Polyamide-6 Composites. Polymers.

[13] Zolfaghari A., Zhang L., Zhou W., Allen Y.Y. (2021). Replication of plastic microlens arrays using electroforming and precision compression molding. Microelectron. Eng..

[14] Wang J., Yi P., Deng Y., Peng L., Lai X., Ni J. (2017). Recovery behavior of thermoplastic polymers in micro hot embossing process. J. Mater. Process. Technol..

[15] Yun D., Kim J., Kim M., Kim D.Y., Hwang J. (2018). Impact print-type hot embossing process technology. Adv. Eng. Mater..

[16] Li K., Xu G., Huang X., Xie Z., Gong F. (2019). Manufacturing of Micro-Lens Array Using Contactless Micro-Embossing with an EDM-Mold. Appl. Sci..

[17] Chang C.Y., Tsao R.H., Wang C.Y. (2020). Novel multilayered hot embossing process for fabricating a microstructure pattern on various polymer substrates. J. Micromech. Microeng..

[18] Guo G., Kethineni C. (2020). Direct injection molding of hybrid polypropylene/wood-fiber composites reinforced with glass fiber and carbon fiber. Int. J. Adv. Manuf. Technol..

[19] Çoğun F., Yıldırım E., Sahir Arikan M.A. (2017). Investigation on replication of microfluidic channels by hot embossing. Mater. Manuf. Process..

[20] Moon I.Y., Lee H.W., Oh Y.S., Kim S.J., Kang S.H. (2019). Characterization of microfibril development on PTFE surface during hot imprinting process and its application for oil–water separation. Int. J. Adv. Manuf. Technol..

[21] Ristok S., Roeder M., Thiele S., Hentschel M., Guenther T., André Z., Herkommer A.M., Giessen H. (2020). Mass-producible micro-optical elements by injection compression molding and focused ion beam structured titanium molding tools. Opt. Lett..

[22] Deshmukh S.S., Goswami A. (2020). Recent developments in hot embossin—A review. Mater. Manuf. Process..

[23] Calaon M., Tosello G., Garnaes J., Hansen H.N. (2017). Injection and injection-compression moulding replication capability for the production of polymer Lab-on-a-Chip with nano structures. J. Micromech. Microeng..

[24] Holmes D.R., Bunn C.W., Smith D.J. (1955). The crystal structure of polycaproamide: Nylon 6. J. Polym. Sci..

[25] Vogelsong D.C. (1963). Crystal structure studies on the polymorphic forms of nylons 6 and 8 and other even nylons. J. Polym. Sci. Part A Polym. Chem..

[26] Zaldua N., Maiz J., de la Calle A., García-Arrieta S., Elizetxea C., Harismendy I., Tercjak A., Müller A.J. (2019). Nucleation and Crystallization of PA6 Composites Prepared by T-RTM: Effects of Carbon and Glass Fiber Loading. Polymers.

